# Human Atrial Fibrillation Is Not Associated With Remodeling of Ryanodine Receptor Clusters

**DOI:** 10.3389/fcell.2021.633704

**Published:** 2021-02-25

**Authors:** Michelle L. Munro, Isabelle van Hout, Hamish M. Aitken-Buck, Ramanen Sugunesegran, Krishna Bhagwat, Philip J. Davis, Regis R. Lamberts, Sean Coffey, Christian Soeller, Peter P. Jones

**Affiliations:** ^1^Department of Physiology and HeartOtago, School of Biomedical Sciences, University of Otago, Dunedin, New Zealand; ^2^Department of Cardiothoracic Surgery, Dunedin Hospital, Dunedin, New Zealand; ^3^Department of Medicine and HeartOtago, Dunedin School of Medicine, University of Otago, Dunedin, New Zealand; ^4^Living Systems Institute, University of Exeter, Exeter, United Kingdom

**Keywords:** atrial fibrillation, calcium channels, dSTORM, ryanodine receptor, super-resolution

## Abstract

The release of Ca^2+^ by ryanodine receptor (RyR2) channels is critical for cardiac function. However, abnormal RyR2 activity has been linked to the development of arrhythmias, including increased spontaneous Ca^2+^ release in human atrial fibrillation (AF). Clustering properties of RyR2 have been suggested to alter the activity of the channel, with remodeling of RyR2 clusters identified in pre-clinical models of AF and heart failure. Whether such remodeling occurs in human cardiac disease remains unclear. This study aimed to investigate the nanoscale organization of RyR2 clusters in AF patients – the first known study to examine this potential remodeling in diseased human cardiomyocytes. Right atrial appendage from cardiac surgery patients with paroxysmal or persistent AF, or without AF (non-AF) were examined using super-resolution (dSTORM) imaging. Significant atrial dilation and cardiomyocyte hypertrophy was observed in persistent AF patients compared to non-AF, with these two parameters significantly correlated. Interestingly, the clustering properties of RyR2 were remarkably unaltered in the AF patients. No significant differences were identified in cluster size (mean ∼18 RyR2 channels), density or channel packing within clusters between patient groups. The spatial organization of clusters throughout the cardiomyocyte was also unchanged across the groups. RyR2 clustering properties did not significantly correlate with patient characteristics. In this first study to examine nanoscale RyR2 organization in human cardiac disease, these findings indicate that RyR2 cluster remodeling is not an underlying mechanism contributing to altered channel function and subsequent arrhythmogenesis in human AF.

## Introduction

The contraction of the heart relies on the highly regulated movement of Ca^2+^ within the cardiomyocytes. The ryanodine receptor (RyR2) is the channel responsible for releasing Ca^2+^ from the sarcoplasmic reticulum (SR) which is required to generate the Ca^2+^ transient and trigger cardiac contraction (Bers, 2002). During excitation-contraction coupling (EC coupling), the action potential facilitates a small influx of extracellular Ca^2+^ via the L-Type Ca^2+^ channel (LTCC), which in turn triggers opening of RyR2 and subsequent Ca^2+^ release from the SR (calcium-induced calcium release, CICR) (Fabiato, 1983). In the healthy heart, this process is tightly controlled on a beat-to-beat basis to maintain a regular heartbeat. Abnormal Ca^2+^ signaling within cardiomyocytes has been implicated in a number of cardiovascular diseases, often including altered function of RyR2 (Wehrens and Marks, 2003; Chakraborty et al., 2019).

The spontaneous release of Ca^2+^ by RyR2 (Ca^2+^ leak) has been shown to contribute to arrhythmogenesis, including in heart failure (HF) and atrial fibrillation (AF) (Voigt et al., 2012; Denham et al., 2018). One of the difficulties in assessing Ca^2+^ leak in human myocytes is the variable yield from enzymatic isolation experiments (Voigt et al., 2015). Previous studies assessing Ca^2+^ transients in myocytes isolated from human atrial tissue demonstrate an average of 1–2 cells per patient as well as relatively high basal spark rates in ‘control’ patient cells compared to diseased animal models of arrhythmia (Knollmann et al., 2006; Voigt et al., 2012, 2014; Zhang et al., 2018; Fakuade et al., 2020). These findings indicate that the viability of these patient cells may be highly variable and subsequently not reliable for direct Ca^2+^ leak assessment at the cellular level. However, studies using isolated channels from human atrial cardiomyocytes reveal significant alterations to RyR2 function in AF patients (Voigt et al., 2012, 2014). These findings, together with numerous studies from animal models of AF, collectively provide a clear demonstration of enhanced RyR2 activity as an underlying basis of arrhythmogenic events in AF. Several mechanisms have subsequently been proposed as contributing to enhanced RyR2 activity in AF, including increased SR load (Voigt et al., 2014) and post-translational modification of RyR2, such as phosphorylation and oxidation which increase channel open probability (Xiao et al., 2007; Neef et al., 2010; Voigt et al., 2012; Xie et al., 2015; Waddell et al., 2016). In addition, the organization of RyR2 channels throughout the cardiomyocyte may influence arrhythmogenicity (Macquaide et al., 2015).

RyR2 channels form clusters within the junctional membrane of the SR (Soeller et al., 2007), typically tightly aligned to the z-disk. In addition to the known direct functional effect on channel open probability, recent work by Asghari et al. (2020) has revealed that phosphorylation of RyR2 can alter the arrangement of channels within clusters. Changes in RyR2 cluster organization have been proposed to be a mechanism by which functional properties of the channel are altered, including the suggestion that smaller clusters have an increased propensity for Ca^2+^ leak (Sobie et al., 2006; Cheng and Lederer, 2008; Jones et al., 2018). With each individual RyR2 channel being ∼30 × 30 nm in size (Yin and Lai, 2000), the ability to detect nanoscale changes in RyR2 clustering properties with conventional imaging techniques, such as confocal microscopy, has been hindered by the resolution limit of these imaging systems. However, the development of super-resolution imaging has allowed this barrier to be overcome and enabled improved imaging resolution to be obtained. Direct stochastic optical reconstruction (dSTORM) is one such super-resolution imaging method which, in recent years, has been successfully employed to investigate the nanoscale clustering properties of RyR2 down to ∼30 nm lateral resolution (Baddeley et al., 2009, 2011). Implementation of dSTORM in ventricular cardiomyocytes has revealed that RyR2 can be organized into a wide variety of cluster sizes within a species, and that the clusters themselves can form units termed “super-clusters” (Hou et al., 2015; Munro et al., 2016). These super-clusters are groups of individual clusters that are within a sufficiently small inter-cluster distance of each other such that Ca^2+^ release from one cluster can trigger CICR from a neighboring cluster within the group (Sobie et al., 2006; Macquaide et al., 2015). Therefore, clusters within a super-cluster are potentially functionally coupled, and hence are also termed calcium release units (CRUs). In addition to inter-cluster spacing, the physiological Ca^2+^ handling by RyR2 channels can be impacted by the packing, or density, of channels within individual clusters (termed RyR2 density in this study). A reduction in the density of RyR2 channels within a cluster would increase the spacing between individual channels and thereby reduce the potential for RyR2–RyR2 interactions, impacting coupled gating properties and channel open probability (Walker et al., 2015).

Recently, super-resolution imaging studies have revealed that RyR2 clusters undergo significant nanoscale remodeling in the failing ventricle of pre-clinical models. This includes the appearance of smaller clusters with reduced RyR2 density, and increased occurrence of “orphaned” clusters (Kolstad et al., 2018; Sheard et al., 2019), which are not associated with LTCC localization. Reduced inter-cluster distances and increased ‘fragmentation’ of CRUs (increased number of individual RyR2 clusters per CRU) are also observed in HF (Kolstad et al., 2018; Sheard et al., 2019), with all of these remodeling properties associated with pro-arrhythmic Ca^2+^ handling of RyR2 (Louch et al., 2013; Walker et al., 2015). In addition to inter-cluster distances, the distribution of RyR2 clusters in relation to the z-disk is an indicator of arrhythmogenic potential in cardiomyocytes. The reduced alignment of clusters to the z-disk, i.e., more clusters between z-disks (termed z-disk dispersion), increases the likelihood of Ca^2+^ leak propagation between sarcomeres to promote pro-arrhythmic Ca^2+^ activity (Macquaide et al., 2015). A reduction in RyR2 associated with the z-disk has also been shown to occur in the failing human heart (Crossman et al., 2011).

While HF is associated with an increased occurrence of arrhythmic activity, AF is the most common form of cardiac arrhythmia, affecting 3% of the adult population (Ball et al., 2013; Kirchhof et al., 2016). It is associated with high mortality and morbidity due to an increased risk of embolism and stroke in patients. Recently, Macquaide et al. (2015) examined RyR2 clustering properties in a long-term paced sheep model of persistent AF using another form of super-resolution imaging – stimulated emission depletion (STED), which achieved ∼60 nm lateral resolution. Although overall RyR2 cluster size remained unchanged, nanoscale RyR2 remodeling was observed in the AF sheep atria, with reduced inter-cluster distances, CRU fragmentation and increased z-disk dispersion (Macquaide et al., 2015). While this study elegantly demonstrated pro-arrhythmic RyR2 remodeling was induced by intra-atrial pacing in sheep heart, the etiology of human AF is heterogeneous in nature (Mackstaller and Alpert, 1997; Cheniti et al., 2018), and may therefore undergo distinct remodeling processes. Moreover, different forms of AF have been clinically characterized in patients. In paroxysmal AF there is a periodic occurrence of AF over a limited timeframe, which typically progresses into a more sustained arrhythmia in the form of persistent AF (Kirchhof et al., 2016).

In the present study we have used dSTORM imaging to investigate the nanoscale organization of RyR2 clusters in human AF. We assessed RyR2 cluster morphology including size, RyR2 density, super-cluster organization and z-disk alignment, and hypothesized that cluster remodeling similar to that reported in the AF sheep atria and in HF would be observed. To our surprise, all RyR2 clustering properties were remarkably unaltered in atrial tissue from patients with either persistent or paroxysmal AF, compared to non-AF. This indicates that, unlike animal models of AF and HF, RyR2 cluster remodeling is not an underlying mechanism driving pro-arrhythmic activity of the Ca^2+^ channel in human AF.

## Materials and Methods

### Patient Selection and Tissue Collection

Following written, informed consent, samples of right atrial appendage (RAA) were collected from patients undergoing coronary artery bypass graft (CABG) surgery at Dunedin Hospital. This study was performed with ethical approval from the New Zealand Human and Disabilities Ethics Committee (LRS/12/01/001) and conformed with the principles outlined in the Declaration of Helsinki. Prior to surgery, all patients underwent transthoracic echocardiography with images acquired by professional echosonographers and reviewed by cardiologists, with functional parameters determined as previously described (Lamberts et al., 2014), including ejection fraction (EF) and E/e′. Attempts were made to keep patient characteristics similar between groups, with age-matched patients selected for this study, excluding those with reduced ejection fraction (EF < 50%) or diastolic dysfunction (E/e′ > 15.0) (Ommen et al., 2000; Nagueh et al., 2016). Measures of left and right atrial chamber size were also recorded and normalized to body surface area (BSA; calculated using the Du Bois formula; Du Bois and Du Bois, 1916), specifically left and right atrial end systolic volume (LAESV and RAESV, respectively).

During the CABG procedure, RAAs were removed prior to cross-clamping and were immediately placed in modified Krebs-Henseleit buffer (in mM: 118.5 NaCl, 4.5 KCl, 0.3 NaH_2_PO_4_, 1.0 MgCl_2_⋅6H_2_O, 25 NaHCO_3_, 11 glucose, 0.5 CaCl_2_ and 6.25 2,3-butanedione monoxime, BDM) which had been previously carbogenated (95% O_2_, 5% CO_2_). The RAA samples were transported to the laboratory within 5–10 min of collection, with tissue pieces flash-frozen in liquid nitrogen and stored at −80°C until use, as previously described (Lamberts et al., 2014). RAA samples were collected from non-diabetic patients pre-operatively diagnosed with either persistent AF or paroxysmal AF, or with no known AF (non-AF). Non-AF patients that developed AF occurrence during the post-operative monitoring period were excluded from the study. The minimum number of patients included in each group was determined by previous group numbers reported in a pre-clinical AF sheep model by Macquaide et al. (2015).

### Immunohistochemistry and Image Acquisition

Right atrial appendage tissue samples were used for immunohistochemistry experiments, adapted from previously described methods (Hou et al., 2015; Munro et al., 2016, 2018). 10 or 20 μm cryosections were cut from the frozen RAA tissue samples and collected onto 1.5 size coverslips coated with poly-L-lysine for immunohistochemistry for super-resolution or confocal imaging, respectively. Sections were allowed to air dry for 1–2 h and then briefly hydrated in PBS before fixation with 2% paraformaldehyde (PFA) for 10 min at room temperature. Samples were triple washed in PBS then permeabilized with 0.1% (confocal) or 1% (super-resolution) triton-X100 for 10 min before blocking with 10% normal goat serum + 0.3 M glycine for 1 h at room temperature. Rabbit anti-RyR2 1:100 (Sigma, HPA020028) and mouse anti-α-actinin 1:200 (Sigma, A7811) were applied overnight at 4°C, followed by incubation with highly cross-adsorbed goat anti-rabbit Alexa Fluor 680 and goat anti-mouse Alexa Fluor 750 for super-resolution, or goat anti-rabbit Alexa Fluor 488 and goat anti-mouse Alexa Fluor 568 for confocal imaging. All secondary antibodies (Life technologies, NZ, United States) were used at 1:200 dilution, incubated for 2 h at room temperature. Confocal samples were co-incubated with wheat germ agglutinin (WGA) Alexa Fluor 647 at 1:200. Coverslips were mounted onto microscope slides for super-resolution imaging with photo-switching buffer containing 90% (v/v) glycerol + 10% glucose (w/v), 10% 10× PBS (v/v) and 5 mM cysteamine (Sigma), pH ∼7.5 and imaged immediately, or ProLong Gold (Life Technologies) and allowed to cure for at least 48 h for confocal imaging.

Confocal imaging was performed on an inverted Nikon A1 + laser scanning microscope with a Nikon 60× oil immersion 1.4 NA objective and captured using a Nikon DS-iR2 color CMOS camera with Nikon NIS Elements software (Nikon, Japan). A total of 488, 561, and 640 nm laser lines were used to excite Alexa Fluor 488, 568 and 647, respectively. Super-resolution imaging (dSTORM) was performed on an Olympus IX81 inverted microscope using an Olympus 60× oil immersion 1.45 NA TIRF objective. A 671 nm laser excitation was used at a highly inclined angle to record single molecule events from the samples on a uEyE CMOS camera (IDS, Germany). Each image was acquired as a series of ∼20–23,000 raw frames at 50 ms/frame with image acquisition, event localization and grayscale rendering performed using custom-written Python Microscopy Environment software^1^. This produced a rendered grayscale 5 × 5 nm/pixel 16-bit TIFF image, in which the pixel intensity is proportional to the density of local events (Baddeley et al., 2010).

### Image Analysis

Analysis of cardiomyocyte size was performed on confocal images of triple-labeled samples using ImageJ. dSTORM event localization and grayscale rendering was performed using custom-written Python Microscopy Environment software (see text footnote 1). Analysis of nanoscale RyR2 clustering properties from dSTORM images was performed based on methods established in previous super-resolution imaging studies (Hou et al., 2015; Munro et al., 2016) using custom Python scripts and ImageJ. All analysis was performed while blinded to patient group allocations. Detailed methods for image analysis are provided in the Supplementary Material.

### Statistical Analysis

Differences between groups were compared using either chi-square, one-way ANOVA with *post hoc* multiple comparisons or Kruskal–Wallis *k*-tests in instances of non-normal distribution of data or Brown-Forsythe and Welsh ANOVA where there was unequal variance between the groups. Correlative data were analyzed using Pearson correlation tests. All analyses were performed using Prism software, v8.0.2 (GraphPad), with a *p*-value of <0.05 considered statistically significant. Data are presented as mean ± SEM for grouped patient analyses, or as scatterplots of individual patient values for correlation analyses. Specific statistical tests used are reported in corresponding figure legends.

## Results

### Clinical Characteristics

Patient characteristics are summarized in Table 1. There were no differences in the mean age, proportion of male/females included or body mass index (BMI) between the three patient groups. Echocardiography parameters representing measures of systolic function and diastolic filling (EF and E/e′, respectively) did not differ between patient groups, however some AF patients were missing E/e′ values due to being in non-sinus rhythm at the time of assessment. Patients with persistent AF did have significantly dilated right atria (RA; 50.4 ± 4.1 mL m^–2^) compared to both non-AF and paroxysmal AF, with RAESV increased ∼2.7- and 1.8-fold, respectively (non-AF: 18.3 ± 1.4 mL m^–2^, paroxysmal AF: 28.1 ± 4.7 mL m^–2^; *p* = 0.0001 and *p* = 0.0074 vs persistent AF, respectively). Significant dilation of the left atria was also observed in persistent AF patients compared to non-AF. There were similar proportions of patients receiving Ca^2+^ blockers, ACE inhibitor and statin medications across the three groups. There was a significant difference in the proportion of patients receiving beta-blockers between the three groups (*p* = 0.0414), with the percentage of patients on this class of medication ranging from 50% (non-AF) to 100% (paroxysmal AF).

**TABLE 1:** Patient characteristics. Summary of patient details including age, gender, body mass index (BMI), ejection fraction (EF), E/e′, right atrial end systolic volume (RAESV), and medication history at time of tissue collection.

**Parameter**	**Non-AF**	**Paroxysmal**	**Persistent**	***p*-value**
Age (years)	71.12.6	71.33.0	74.32.3	0.6662
Gender (M/F)	7/3	6/3	6/2	0.9314
BMI (kg m^–2^)	27.30.6	28.90.7	28.40.4	0.1552
EF (%)	60.01.2	60.71.9	55.71.4	0.0736
E/e′	10.11.0	12.12.2	13.90.1	0.4630
RAESV (mL m^–2^)	18.31.4	28.14.7	50.44.1^###,††^	< 0.0001***
LAESV (mL m^–2^)	35.44.4	50.310.7	91.215.8^‡‡^	0.0027**
**Medications**				
Ca^2+^ channel blocker	2/10	3/9	2/8	0.8011
ACE inhibitor	5/10	3/9	3/8	0.7429
Beta blocker	5/10	9/9	6/8	0.0457*
Statin	9/10	6/9	6/8	0.4624
# patients	10	9	8	

Age, BMI and EF analyzed by one-way ANOVA with *post hoc* Tukey’s multiple comparisons where applicable. RAESV was analyzed by Brown-Forsythe and Welsh ANOVA. Gender and medication distributions analyzed by Chi-squared contingency analysis. E/e′ and LAESV were analyzed with Kruskal–Wallis test; some patient values missing for E/e′ (non-AF *n* = 9, paroxysmal AF *n* = 7, persistent AF *n* = 2). * *p* < 0.05, ** *p* < 0.01, *** *p* < 0.001 between groups. ^###^*p* = 0.0001 non-AF versus persistent AF. ^††^*p* = 0.0077 paroxysmal AF versus persistent AF. ^‡‡^*p* = 0.0021 non-AF versus persistent non-AF.

### Atrial Remodeling in Human AF

**FIGURE 1:**
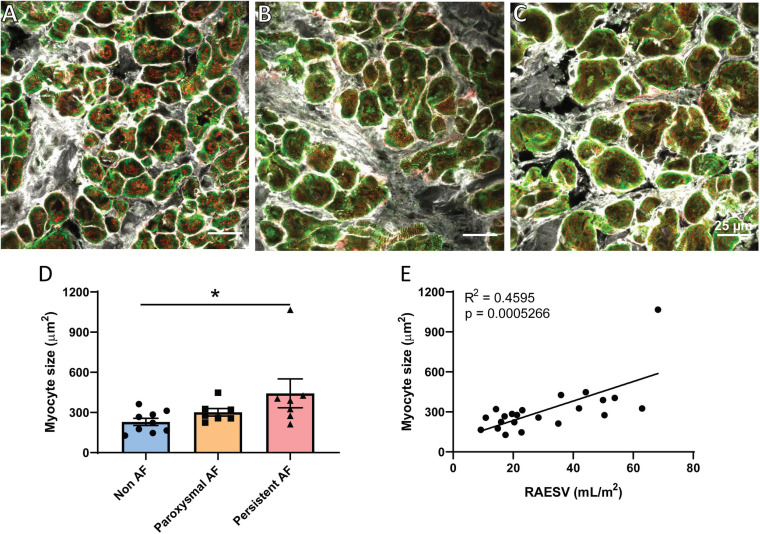
Cardiomyocyte hypertrophy in human RAA with persistent AF. Confocal micrographs of triple labeled RAA tissue samples from **(A)** non-AF, **(B)** paroxysmal AF, and **(C)** persistent AF patients, showing RyR2 (red), α-actinin (green) and WGA (gray) staining in transverse-orientation cardiomyocytes. Scale bar = 25 μm. **(D)** Mean cardiomyocyte cross-sectional area in the three patient groups, which **(E)** significantly correlates with RA volume across all patients. **(D)** Data displayed as mean ± SEM; analyzed by Kruskal–Wallis *k*-test; non-AF *n* = 9; paroxysmal AF *n* = 7; persistent AF *n* = 7. Correlation analyzed by Pearson’s correlation analysis; total *n* = 22. *p < 0.05 persistent AF versus non-AF.

One of the hallmark structural changes in AF is dilation of the atria (Eckstein et al., 2008; Schotten et al., 2011) as was observed in our cohort of persistent AF patients (Table 1). Atrial cardiomyocyte hypertrophy has also previously been described in both human and animal models of persistent AF (Ausma et al., 1997; Schotten et al., 2011) and was assessed in the three patient groups using confocal imaging. Triple-labeled sections of cardiomyocytes in transverse orientation from the three patient groups were used to measure cardiomyocyte cross-sectional area to assess potential cellular hypertrophy (Figures 1A–C). Analysis revealed significant hypertrophy of RAA cardiomyocytes in the persistent AF patients compared to non-AF (Figure 1D; *p* = 0.0374). Cardiomyocyte size in the paroxysmal AF RAA was not significantly different from the other two patient groups. Interestingly, the degree of RA dilation significantly correlated with RAA cardiomyocyte hypertrophy when assessed across all patient groups (Figure 1E; *R*^2^ = 0.4595, *p* = 0.0005). Given the remodeling observed at both the chamber and cellular level in human AF, we proceeded to investigate if additional nanoscale protein changes are also observed in these patients.

### Individual RyR2 Cluster Properties in Human AF

**FIGURE 2:**
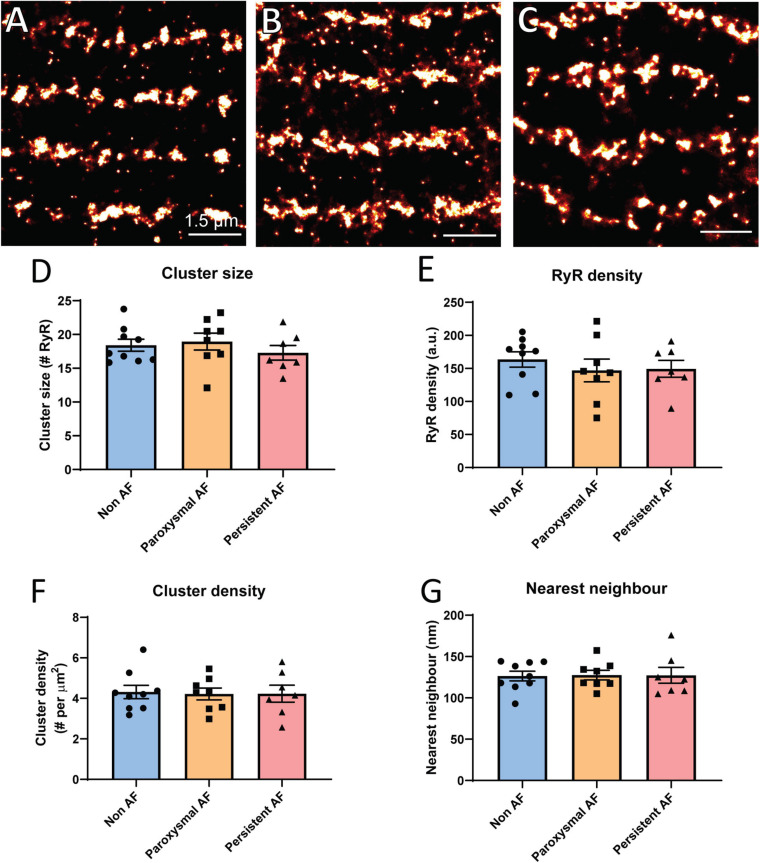
Individual RyR2 clusters do not remodel in human AF. Rendered super-resolution (dSTORM) images of RyR2 clusters in human RAA tissue from **(A)** non-AF, **(B)** paroxysmal AF and **(C)** persistent AF patients. Scale bar = 1.5 μm. Image analysis reveals the mean **(D)** RyR2 cluster size, **(E)** intra-cluster RyR2 packing density, **(F)** cluster density per area cardiomyocyte, and **(G)** nearest neighbor distance for the three patient groups. Data displayed as mean ± SEM. Analysis performed using one-way ANOVA; non-AF *n* = 9, paroxysmal AF *n* = 8, and persistent AF *n* = 7.

Based on the reported overall remodeling of RyR2 in a sheep model of persistent AF (Macquaide et al., 2015), it was of interest to determine if similar remodeling occurs in atrial tissue from AF patients using dSTORM. Rendered super-resolution images revealed no visible difference in the overall morphology of RyR2 clusters in either of the AF patient groups compared to non-AF patient RAA samples (Figures 2A–C). To verify this observation, analyses of nanoscale clustering properties were performed with various morphological parameters assessed. Analysis revealed a mean cluster size of 18.41 ± 0.89 RyR2 channels in RAA from non-AF patients, and confirmed this did not differ in either of the AF patient groups (Figure 2D; paroxysmal AF: 18.95 ± 1.26 RyR2; persistent AF: 17.29 ± 1.08 RyR2). Furthermore, the cumulative frequency of RyR2 clusters as a function of size was unchanged in the three patient groups (see Supplementary Figure S2). This also revealed that the majority of clusters were small in size, with a median cluster size of ∼4 channels, and 50% of clusters being <4–5 RyR2 channels in size in all patient groups.

While the exact role of RyR2 cluster size on Ca^2+^ release remains unclear, it has been demonstrated that the packing density of individual RyR2 channels within a cluster (inter-channel distance) is important for channel opening properties (Walker et al., 2015). We therefore also assessed RyR2 labeling density *within* individual clusters. RyR2 density did not significantly differ between the patient groups (non-AF: 163.6 ± 11.6 a.u., paroxysmal AF: 146.9 ± 17.2 a.u. and persistent AF: 149.4 ± 12.9 a.u.; Figure 2E), indicating that RyR2 channel packing is unaltered within clusters in the RAA of AF patients.

Additional analysis was performed to assess the overall density of clusters present throughout the cardiomyocyte, as measured by the number of clusters per area unit. This was also found to be unchanged in the AF patient groups compared to non-AF, with a mean of ∼4.3 clusters per μm^2^ (Figure 2F). Given the evidence of reduced inter-cluster distances in the AF sheep atria and the role this remodeling has in pro-arrhythmic Ca^2+^ propagation (Macquaide et al., 2015), we also assessed nearest neighbor (inter-cluster) distances in the human RAA samples. There were no differences in the mean nearest neighbor distances across the three patient groups, with values of 126.4 ± 5.9 nm in non-AF, 127.6 ± 5.8 nm in paroxysmal AF and 127.3 ± 9.6 nm in persistent AF (Figure 2G). Together, these findings demonstrate that there is no evidence of individual RyR2 cluster remodeling within the RAA of AF patients.

### RyR2 Super-Cluster Organization in Human AF Atria

**FIGURE 3:**
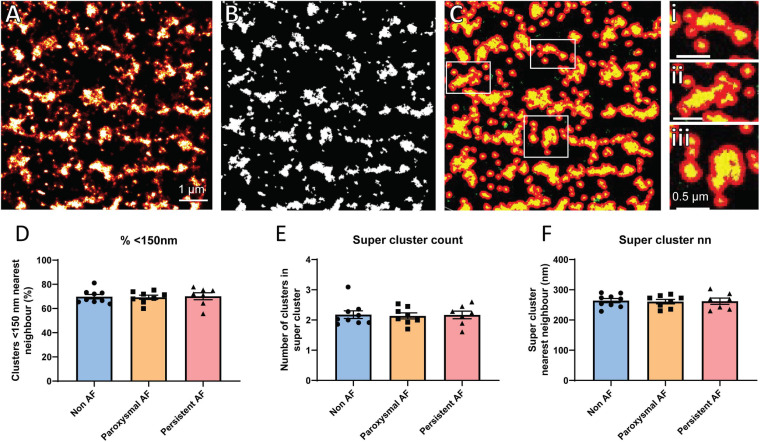
RyR2 super-cluster properties are unaltered in human AF. Demonstration of super-cluster analysis performed on rendered dSTORM images of RyR2 clusters in a non-AF patient RAA, including **(A)** original rendered image, **(B)** mask generated after application of threshold, and **(C)** detection of super-clusters, with examples shown in panels i–iii. **(A–C)** Scale bar = 1 μm; panels i–iii scale bar = 0.5 μm. Analysis reveals the mean **(D)** percentage of clusters within 150 nm of nearest neighbor, **(E)** number of clusters in a super-cluster, and **(F)** super-cluster nearest neighbor distance in the RAA of non-AF, paroxysmal AF, and persistent AF patients. Data displayed as mean ± SEM. All analyses performed with one-way ANOVA; non-AF *n* = 9, paroxysmal AF *n* = 8, persistent AF *n* = 7.

Several studies have previously demonstrated that the functional grouping of RyR2 clusters into super-clusters (also termed CRUs) plays an important role in the propensity for pro-arrhythmic Ca^2+^ activity, and is altered in the atria of AF sheep (Macquaide et al., 2015). Therefore, the organization of RyR2 clusters into super-cluster (groups of individual clusters < 150 nm apart) was assessed in the human RAA. To achieve this, rendered dSTORM images had a threshold applied to enable generation of a binary mask, with Euclidean distances determined such that clusters within 150 nm of each other were grouped together into super-clusters (Figures 3A–C). From this analysis, it was revealed that ∼70% of RyR2 clusters were within 150 nm of their nearest neighboring cluster in non-AF RAA cardiomyocytes, which was unchanged in either of the AF patient groups (non-AF: 69.78 ± 1.84%; paroxysmal AF: 69.26 ± 1.76%; persistent AF: 70.15 ± 2.90%, respectively; Figure 3D). While this finding was not surprising given the absence of differences in nearest neighbor distance reported in the patient groups, it remained of interest to ascertain whether there was potential remodeling of the super-clusters themselves in human AF. There were no differences in the mean number of RyR2 clusters contained within super-clusters in the RAA across the three patient groups, indicating that CRU fragmentation is not occurring in these AF samples. Mean values for patient groups were non-AF: 2.18 ± 0.13, paroxysmal AF: 2.14 ± 0.10 and persistent AF: 2.17 ± 0.13 clusters per super-cluster (Figure 3E). Furthermore, the mean nearest neighbor distance between super-clusters in non-AF of 264.1 ± 7.0 nm did not differ to the 260.9 ± 7.3 nm and 262.3 ± 10.4 nm measured in paroxysmal and persistent AF samples, respectively (Figure 3F). Together, these analyses indicate that both the properties of individual RyR2 clusters and their functional grouping into super-clusters are remarkably unaltered in the RAA of AF patients. A summary of RyR2 cluster morphological characteristics for each patient group are presented in Table 2.

**TABLE 2:** Nanoscale RyR2 clustering properties in human RAA.

**Parameter**	**Non-AF**	**Paroxysmal AF**	**Persistent AF**	***P*-value**
Cluster size (# RyR2)	18.410.89	18.951.26	17.291.08	0.5719
Cluster density (# per μm^2^)	4.310.33	4.220.29	4.230.42	0.9754
RyR packing density (a.u.)	163.611.6	146.917.2	149.412.9	0.6530
Nearest neighbor (nm)	126.45.9	127.65.8	127.39.6	0.9916
Clusters < 150 nm (%)	69.781.84	69.261.76	70.152.90	0.9596
Super-cluster nearest neighbor (nm)	264.17.0	260.97.3	262.310.4	0.9594
Clusters in super-cluster (#)	2.180.13	2.140.10	2.170.13	0.9672
# clusters analyzed	23,112	22,968	15,007	
# images(# patients)	32(9)	30(8)	23(7)	

*Summary of RyR2 cluster parameters measured from rendered dSTORM images of RAA samples from non-AF, paroxysmal AF and persistent AF patients. Parameters for individual clusters include cluster size and density, RyR2 packing density and nearest neighbor distance. Super-cluster analysis measurements include the percentage of clusters within 150 nm of nearest neighbor, distance between nearest neighboring super-clusters and the number of clusters in a super-cluster. The total number of individual clusters and images analyzed in each patient group is indicated. All analyses performed with one-way ANOVA. Data presented as mean ± SEM.*

### Absence of z-Disk Dispersion in Human AF

**FIGURE 4:**
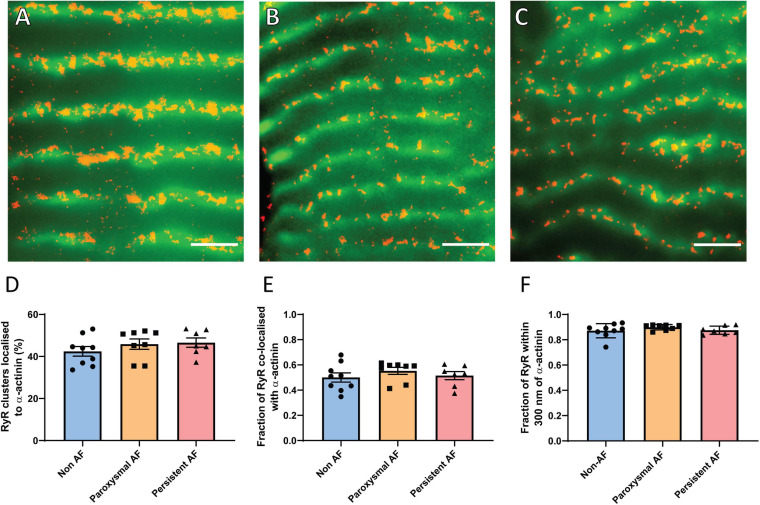
RAA cardiomyocytes in AF patients do not demonstrate z-disk dispersion. Dual immunolabeling of RyR2 (red, dSTORM) and α-actinin (green, widefield) in RAA tissue of **(A)** non-AF, **(B)** paroxysmal AF and **(C)** persistent AF patients. Scale bar = 2 μm. Image analysis reveals no change in the extent of z-disk dispersion of RyR2 clusters based on **(D)** the percentage of clusters localized to the z-disk, the fraction of total RyR2 labeling **(E)** co-localized with or **(F)** within 300 nm of α-actinin across the three patient groups. Data displayed as mean ± SEM. Analysis performed with Kruskal–Wallis *k*-test; non-AF *n* = 9, paroxysmal AF *n* = 8, persistent AF *n* = 7.

In addition to the distribution of RyR2 clusters in relation to each other, their localization throughout the cardiomyocyte is also an indicator of arrhythmogenic potential. The redistribution of clusters away from the normal z-disk alignment and toward inter-sarcomeric regions, known as z-disk dispersion, effectively promotes pro-arrhythmic Ca^2+^ propagation (Macquaide et al., 2015). Such dispersion has been reported to occur in a sheep model of AF (Macquaide et al., 2015), and was therefore also examined in the human AF samples. The percentage of individual RyR2 clusters localized to the z-disk (as determined by α-actinin labeling) was assessed in the three patient groups (Figures 4A–C). In non-AF RAA, there was a mean of 42.44 ± 2.28% of RyR2 clusters aligned with the z-disk, which was not significantly altered in either of the AF patient groups (paroxysmal AF: 45.89 ± 2.45% and persistent AF: 46.60 ± 2.24%; Figure 4D).

While the overall proportion of clusters aligned to the z-disk was unchanged in AF, it is possible that clusters of different sizes are differentially distributed throughout the cardiomyocytes in AF (e.g., larger clusters aligned to z-disk, smaller clusters between sarcomeres). Therefore, the fraction of RyR2 labeling (as an indicator of the proportion of channels) associated with α-actinin was also measured to determine whether the distribution of RyR2 channels across the cardiomyocyte was altered in the AF patient groups. This involved measuring the fraction of total RyR2 labeling either co-localized with, or within 300 nm of α-actinin. Analyses revealed that, on average, 50.0 ± 3.6% of RyR2 labeling was co-localized with α-actinin in the non-AF RAA, with 87.2 ± 1.9% localized within 300 nm of the z-disk (Figures 4E,F). This indicates that the majority of RyR2 channels are localized either at, or in close proximity to the z-disk. In RAA tissue of patients with paroxysmal AF, these values were unchanged, with 55.3 ± 2.8% and 89.9 ± 0.8% of RyR2 labeling co-localized with or within 300 nm of α-actinin, respectively. Persistent AF patient samples also did not differ from either of the other patient groups, with 51.5 ± 3.2% of RyR2 labeling co-localized with, and 87.5 ± 1.2% of RyR2 labeling within 300 nm of α-actinin. Combined, these data indicate that z-disk dispersion of RyR2 channels/clusters does not contribute to pro-arrhythmic Ca^2+^ activity in the RAA of human AF patients.

### Patient Characteristics Do Not Correlate With Cluster Remodeling

Epidemiology studies have consistently identified age as an independent risk factor for the development of AF in patients (Benjamin et al., 1994; Stewart et al., 2001). In order to determine if the inter-patient variability observed in RyR2 cluster morphology parameters within groups was attributed to patient characteristics, such as age, correlation analyses were performed across all patient groups. From these analyses, it was determined that no significant relationships existed between RyR2 clustering properties and patient characteristics (see Supplementary Figure S3). There were also no significant differences in correlative relationships between groups when analyzed based on AF status (see Supplementary Figure S4). Cardiomyocyte size did not correlate with RyR2 clustering properties (see Supplementary Figure S5).

## Discussion

It has previously been demonstrated that single molecule localization imaging techniques are able to provide vastly superior resolution to that in standard fluorescent methods, such as confocal line-scanning microscopy, with dSTORM enabling lateral resolution of ∼30 nm to be achieved (Baddeley et al., 2009, 2011). Using this latter imaging technique, we have assessed the nanoscale morphology and organization of RyR2 clusters in RAA tissue from patients with persistent and paroxysmal AF, or without AF. This is, to the best of our knowledge, the first study to examine RyR2 clustering properties in human cardiac disease using dSTORM. Consistent with previous studies, gross atrial and cellular remodeling was identified in the persistent AF patients, with our findings also revealing that RyR2 clustering properties of both individual clusters and super-clusters (CRUs) remain remarkably unaltered in human AF. This suggests that pre-clinical models may not represent the nanoscale organization of RyR2 occurring in human AF tissue.

### Individual RyR2 Clusters Do Not Remodel in Human AF

We identified that, on average, the RyR2 clusters in human RAA cardiomyocytes can contain ∼18 RyR2 channels in patients with no known AF. While it has been demonstrated that the size of an “average” cluster can vary between species (Franzini-Armstrong et al., 1999), this value is not dissimilar to that of ∼15 RyR2 per cluster reported by Macquaide et al. (2015) in the sheep atria.

Previous research into the effect of cluster morphology on the functional properties of RyR2 suggested that smaller clusters have an increased propensity for diastolic leak due to reduced channel stability from fewer RyR2–RyR2 interactions (Sobie et al., 2006; Cheng and Lederer, 2008). Meanwhile, more recent experimental and modeling work has demonstrated that small RyR2 clusters (<60 RyR2) exhibit a reduced frequency of Ca^2+^ sparks and thereby limit the occurrence of arrhythmogenic Ca^2+^ events (Galice et al., 2018). However, this latter study was based on confocal imaging techniques which would not have been able to resolve very small clusters (<6 RyR2), which potentially contribute to the “rogue” channels previously described to be attributed to diastolic leak (Sobie et al., 2006). Computational modeling work by Xie et al. (2019) suggests that it is the overall variability in cluster sizes within the myocyte which is a key indicator of arrhythmogenic Ca^2+^ activity, with the presence of more heterogeneous cluster sizes increasing the likelihood of Ca^2+^ waves. The importance of this mechanism in cardiac pathologies remains unclear, with nanoscale imaging of RyR2 clusters in a rat model of HF revealing a reduced range of cluster sizes with an overall reduction in mean size (Sheard et al., 2019).

The predicted impact of small RyR2 clusters on diastolic leak, and therefore the susceptibility to arrhythmia, critically depends on the presence or absence of direct coupling between cardiac RyR2 channels, which is currently still a topic of debate in Ca^2+^ signaling physiology. Despite the lack of clarity as to the link between RyR2 cluster size and spontaneous Ca^2+^ release, we identified that mean RyR2 cluster size was unaltered in either persistent or paroxysmal AF samples, and that all three patient groups showed a highly similar distribution of cluster sizes. While smaller clusters have been reported in rodent HF models (Kolstad et al., 2018; Sheard et al., 2019), this does not appear to be a remodeling mechanism shared in AF, with unchanged cluster size also reported by Macquaide et al. (2015) in a sheep model of persistent AF.

Perhaps more relevant to RyR2 function is the density of channel packing within individual clusters. Again, this is a topic of dispute, dependent on the presence of direct coupling mechanisms between channels, with increased inter-channel distances suggested to reduce the propensity for spontaneous Ca^2+^ release through a reduction in the co-operative opening of channels (Marx et al., 2001; Walker et al., 2015; Munro et al., 2016). Conversely, others suggest a reduction in RyR2–RyR2 interactions through larger inter-channel distances could lead to enhanced Ca^2+^ channel opening (Asghari et al., 2020). This may be due to a reduction in steric hinderance to inhibit the physical opening of neighboring channels, which could occur with tightly packed channels (Asghari et al., 2014, 2020). In addition to being smaller in size, RyR2 clusters show a reduction in the channel density, with increased inter-channel distances within clusters in animal models of HF (Kolstad et al., 2018; Sheard et al., 2019).

It has been shown that the signal intensity in these dSTORM rendered images is proportional to the number of protein molecules present within the sample (Baddeley et al., 2009, 2011). By assessing the labeling density within the RyR2 clusters, we can get an indirect measure of the packing density of channels within individual clusters (Munro et al., 2016). Using this method, we found that the RyR2 density within clusters was unchanged in both of the AF patient groups compared to non-AF, suggesting that the packing of channels into clusters is unlikely to be contributing to differences in RyR2 gating properties in human AF.

### Human AF RyR2 Inter-Cluster Properties Are Unchanged

A reduction in inter-cluster (nearest neighbor) distances has been previously demonstrated to promote the propagation of spontaneous Ca^2+^ release across neighboring clusters (Walker et al., 2015). This leads to a greater potential for a critical Ca^2+^ concentration to be reached, triggering delayed after-depolarizations (DADs) and subsequent arrhythmic activity. Such inter-cluster remodeling has indeed been observed in rodent models of HF (Kolstad et al., 2018; Sheard et al., 2019) and a sheep model of persistent AF (Macquaide et al., 2015). Surprisingly, we did not identify a change in the mean nearest neighbor cluster distances in RAA cardiomyocytes of persistent or paroxysmal AF patients compared to non-AF. This indicates that an increased proximity of RyR2 clusters does not contribute to pro-arrhythmic Ca^2+^ properties in human AF.

While inter-cluster distance is an indicator of arrhythmic potential, the organization of neighboring clusters is also considered a significant factor, including the composition of super-clusters, or CRUs. The capacity for functionally coupled clusters localized <150 nm apart from each other to generate pro-arrhythmic Ca^2+^ release has been shown to be increased when these CRUs are more “fragmented,” as observed in animal models of AF (Macquaide et al., 2015) and HF (Kolstad et al., 2018). In particular, the presence of more, smaller clusters contributing to the composition of a CRU was identified as increasing the propagation of spontaneous Ca^2+^ release in AF (Macquaide et al., 2015). Despite the evidence for this remodeling being a mechanism contributing to arrhythmogenic activity in a sheep model of AF, no changes in RyR2 super-cluster properties were identified in the AF patients in this study.

When these findings are considered in the context of the hypertrophy observed in persistent AF, it suggests an adaptive response to maintain RyR2 cluster organization. Despite enlargement of the cardiomyocytes, there is no change to the distribution of clusters within these cells in persistent AF. This indicates a corresponding increase in the total number of clusters such that the density per unit area and inter-cluster distances are maintained, as well as overall distribution throughout the cardiomyocyte.

### Alignment of RyR2 Clusters to the z-Disk Is Unaltered in Human AF

Altered organization of RyR2 clusters in relation to the z-disk was not observed in the human AF cardiomyocytes despite a reduced localization of RyR2 cluster within 300 nm of the z-disk reported a sheep model of persistent AF (Macquaide et al., 2015). However, these latter measurements were performed without visualization of α-actinin, which may explain minor discrepancies in the values obtained compared to the human atria, as it has been demonstrated that z-disks are not linear in organization across the cardiomyocyte (Jayasinghe et al., 2010). The expected localization of RyR2 clusters to the z-disk is based on the well-described observations in ventricular cardiomyocytes of the alignment of the transverse tubules (t-tubules) to the z-disk, where the dyad is formed between the t-tubule and junctional SR membranes. Disorganization of ventricular t-tubules is well characterized in cardiomyopathies (Lyon et al., 2009; Crossman et al., 2015), which contributes to impaired Ca^2+^ handling. However, the organization of t-tubules (and therefore dyads) in the atria is less established, with their presence suggested to be variable both within and between species (Kirk et al., 2003; Richards et al., 2011; Frisk et al., 2014). This includes human atria, in which it has been demonstrated that not all cardiomyocytes contain a distinguishable t-tubule network, and in those that do, there is less apparent organization compared to that observed in the atria of other large mammals (Richards et al., 2011). We observed a similar finding in our human RAA samples, in which a clear t-tubule system was not present in all cardiomyocytes, regardless of AF status (not shown). As previously reported, there was reduced clarity of t-tubule staining in longitudinal orientation compared to transverse (Richards et al., 2011), meaning that the analysis of t-tubule alignment with z-disk/RyR2 was not possible in these samples. Regardless of t-tubule organization in human atria, this study demonstrates that RyR2 clusters are not redistributed from the z-disk in AF to promote arrhythmogenic Ca^2+^ propagation.

### What Mechanisms May Underlie Arrhythmogenicity in the Human AF Atria?

The role of Ca^2+^ mishandling has been well established as an underlying trigger of arrhythmic activity in cardiomyocytes. This includes conclusive demonstration of increased RyR2 mediated Ca^2+^ leak in atrial cardiomyocytes from AF patients, with subsequent development of DADs (Voigt et al., 2012, 2014; Beavers et al., 2013). While the exact mechanism(s) leading to this pathological Ca^2+^ activity in human AF is not fully understood, the present study clearly demonstrates that remodeling of the RyR2 clusters is not a contributing factor; however, potential alternative underlying causes are discussed below.

One of the main suggested mechanisms contributing to Ca^2+^ mishandling in both HF and AF is hyperphosphorylation of RyR2. In particular, several studies demonstrate an upregulation of pS2814 in persistent/chronic AF patients, which increases RyR2 open probability and thereby results in enhanced Ca^2+^ leak (Neef et al., 2010; Voigt et al., 2012). However, this increased post-translational modification is reportedly not found in paroxysmal AF human samples (Voigt et al., 2014), suggesting additional mechanisms are in play. The relative expression of several RyR2-associated proteins (e.g., junctophilin-2, calsequestrin, FK-binding protein) has also been demonstrated to be reduced in paroxysmal and persistent AF patients (Beavers et al., 2013; Xie et al., 2015). While some studies report increased expression of RyR2 in AF patients, this is normalized to calsequestrin expression rather than typical housekeeping proteins (Voigt et al., 2014). As the loss of such accessory proteins from RyR2 is associated with an increase in Ca^2+^ leak, the relative expression ratio of these proteins may be a more important trigger for spontaneous Ca^2+^ release than RyR2 expression or cluster size in the setting of human AF, and warrants further investigation.

Recent computational modeling work by Kolstad et al. (2018) clearly demonstrated that a reduction in inter-cluster distances increases the fidelity of spontaneous Ca^2+^ release propagation to nearby neighboring clusters within a distance that would be considered forming a super-cluster. Interestingly, this was demonstrated to be largely due to increasing the amount of junctional SR “padding” around the cluster, such that there was reduced distance, not only between clusters, but also between regions of junctional SR. It was this latter factor that was identified as more important in determining the chance of inter-cluster Ca^2+^ propagation, rather than spacing between the clusters themselves within the super-cluster (Kolstad et al., 2018). The absence of changes in CRUs identified in the present study suggests that other forms of remodeling are potentially occurring which promote spontaneous Ca^2+^ propagation in AF. It is therefore possible that junctional SR remodeling is playing a key role in promoting arrhythmogenicity in AF rather than the apparent organization of RyR2 super-clusters.

### Comparison to Pre-Clinical Sheep Model of AF

The most probable cause for the disparity in findings between the present study and those reported in the sheep model of AF is the differences in etiology of the arrhythmia. While CRU remodeling appears robust when driven by tachy-pacing in the sheep atria (Macquaide et al., 2015), this pathological stimulus is not fully representative of the factors driving arrhythmia in the human atria, which are more heterogenous in nature (Mackstaller and Alpert, 1997; Calvo et al., 2018). Although tachycardia may be involved in some instances of human AF (or in the role of progression to heart failure; Sugumar et al., 2019), there are several additional pathological triggers and substrates involved, including hypertension and atrial fibrosis (Nattel, 2017; Cheniti et al., 2018). Significant atrial dilation is characteristic in AF patients (Mackstaller and Alpert, 1997), with our findings further supporting this observation; however, the degree of atrial dilation has not been quantified in the sheep model. These factors are likely to contribute to the variation in idiopathic AF development/progression observed in human AF and may result in an overall different pattern of cellular or molecular remodeling in the atria compared to artificially driven tachy-pacing alone. It is also likely that the changes in clustering properties observed in the pre-clinical sheep model of AF are being driven by the artificial tachy-pacing itself rather than as a result of the sustained AF in the sheep heart, with these two mechanisms difficult to separate in this model.

Species-dependent remodeling mechanisms may also be contributing to the differences in observed the sheep model compared to human AF findings. It has been demonstrated that differences exist in the appearance of t-tubules in the adult atria of different species (Richards et al., 2011), suggesting that the organization of dyads and therefore RyR2 cluster organization may also differ.

### Limitations

One limitation of note for this study was that the non-AF group consisted of patients undergoing cardiac bypass surgery, indicating that these hearts were not completely “healthy.” It is therefore possible that some degree of cardiac remodeling may be present in these samples compared to what would be observed in non-cardiac patients. Unfortunately, it was not possible to obtain non-surgical tissue samples for this study. However, one advantage of the inclusion of non-AF CABG patients as the control group is that they were treated with cardiovascular medications to a similar extent as AF patients (see Table 1), ruling out the potential involvement of pharmacological intervention on RyR2 cluster remodeling as a factor in observed results. Furthermore, not only did the non-AF patients have no prior history of this arrhythmia, but during the 3-day follow up period, they did not present with incidence of post-surgical AF, which is commonly observed in CABG patients. This supports the use of atrial tissue from these patients as a suitable control for comparison of potential arrhythmogenic remodeling in the AF patient tissue.

Further to this is the limitation on the type of tissue utilized, namely from the RAA rather than the left atrium, which is the chamber most associated with AF (Haïssaguerre et al., 1998). Although obtaining left atrial tissue was not possible for this study, ∼30–50% of atrial arrhythmic activity arises from the RA (Hasebe et al., 2016; Li et al., 2016), with biatrial abnormalities commonly observed in AF (Stiles et al., 2009), making the RA an appropriate study sample. Furthermore, our study clearly demonstrates the involvement of the RA in persistent AF, with significant remodeling observed at both the chamber and cellular level. Several studies published by Voigt et al. have demonstrated functional changes occur in myocytes from the RAA of AF patients, including increased spontaneous Ca^2+^ activity resulting from enhanced channel open probability (Voigt et al., 2012, 2014). This indicates Ca^2+^ dysfunction occurs in the RAA, making it suitable for investigating remodeling associated with human AF.

While it is critical to assess potential RyR2 cluster remodeling in clinical samples, it is largely impracticable to directly correlate these structural parameters with Ca^2+^ handling within these tissues. This is predominantly due to typically poor viability and low yield of myocytes isolated from human atrial biopsies. While studies using isolated RAA myocytes from AF patients have been reported, these reveal a high rate of spontaneous Ca^2+^ activity in “control” patient myocytes, compared to equivalent control myocytes from animal models (Knollmann et al., 2006; Voigt et al., 2012, 2014; Zhang et al., 2018). This makes interpretation of any disease mediated changes in Ca^2+^ handling in human samples difficult to interpret reliably. Although this study does not directly assess RyR2 function in the patient cohort, it builds on a vast body of literature linking AF with dysfunctional Ca^2+^ release through RyR2.

## Conclusion

The findings from this study indicate that remodeling of the RyR2 clusters is not responsible for the increased propensity for Ca^2+^ spontaneous leak and arrhythmogenicity previously observed in human AF cardiomyocytes. Unlike in a single-cause (tachy-pacing) model of AF, significant RyR2 cluster remodeling is not observed in human AF, which arises from heterogeneous factors. It is therefore likely that it is either the post-translational modification of RyR2s (e.g., phosphorylation, oxidation, nitrosylation) or the interaction with accessory proteins that is altered and implicated in the changes in RyR2 activity in AF, or a combination of the two. These findings are promising as at this time there seems to be greater potential to develop a therapeutic strategy to target post-translational modifications or protein binding interaction as a method for treating AF, as opposed to altering RyR2 clustering properties which still lack deeper mechanistic understanding.

## Data Availability Statement

The raw data supporting the conclusions of this article will be made available by the authors, without undue reservation.

## Ethics Statement

The studies involving human participants were reviewed and approved by New Zealand Health and Disabilities Ethics Committee. The patients/participants provided their written informed consent to participate in this study.

## Author Contributions

MM performed the experiments and data analysis. IvH collected patient data and surgical samples. RS, KB, and PD provided materials. HA-B, RL, SC, and CS assisted with analysis and data interpretation, and edited the manuscript. MM and PJ designed the study, interpreted the data, and wrote the manuscript. All authors contributed to the article and approved the submitted manuscript.

## Conflict of Interest

The authors declare that the research was conducted in the absence of any commercial or financial relationships that could be construed as a potential conflict of interest.

## Footnotes

1 https://bitbucket.org/david_baddeley/python-microscopy
