# Supplementary material for: Human Atrial Fibrillation Is Not Associated With Remodeling of Ryanodine Receptor Clusters
Source: Front Cell Dev Biol. 2021 Feb 25;9:633704. doi: 10.3389/fcell.2021.633704 (PMC7947344; doi:10.3389/fcell.2021.633704)
Supplement: Supplementary file 1 [file Data_Sheet_1.PDF]

Human atrial fibrillation is not associated with remodeling of ryanodine receptor clusters

Michelle L. Munro[1*], Isabelle van Hout[1], Hamish M. Aitken-Buck[1], Ramanen Sugunesegran[2], Krishna Bhagwat[2], Philip J Davis[2], Regis R. Lamberts[1], Sean Coffey[3], Christian Soeller[4] & Peter P. Jones[1*]

[1]Department of Physiology and HeartOtago, School of Biomedical Sciences, University of Otago, Dunedin, New Zealand;

[2]Department of Medicine and HeartOtago, Dunedin School of Medicine, University of Otago, Dunedin, New Zealand;

[3]Department of Cardiothoracic Surgery, Dunedin Hospital, Dunedin, New Zealand;

[4]Living Systems Institute, University of Exeter, Exeter, United Kingdom.

Supplementary Material

Image analysis

Confocal image analysis was performed using ImageJ. Cardiomyocyte cross-sectional area was assessed in triple labelled tissue sections with cells in transverse orientation, as determined by α-actinin staining. WGA labelling was used to determine the outline of cells, with RyR2 and α-actinin labelling within WGA-free areas used to positively identify cardiomyocytes. The perimeter of these transverse cardiomyocytes was manually traced, with the resulting ROI area determined as the measure for cardiomyocyte size.

Analysis of RyR2 clusters was performed based on methods established in previous dSTORM imaging studies.[1, 2] Following triangulation methods to produce the rendered image (as described in Baddeley et al., 2010[3]), a binary mask was generated by applying an intensity threshold such that 70% of the integrated image intensity was retained for inclusion in the mask. The labelled regions within the mask were analyzed to determine the two-dimensional area of the individual RyR2 clusters. The maximum number of RyR2 channels present within a single cluster was then calculated based on an isotropic 30 nm centre-to-centre packing density of the receptor to give a measure of RyR2 cluster size. The density of RyR2 clusters within the cell (cluster density) was calculated by dividing the total number of clusters present

by the area of cell analyzed. The density of RyR2 labelling within clusters was determined by using the generated mask to locate the clusters as regions of interest (ROIs) within the original rendered dSTORM image (no thresholding), to measure the raw integrated greyscale intensity within each ROI. This intensity value was then divided by the area of the cluster to provide a measure of the RyR2 labelling density within individual clusters, termed RyR2 density.

The inter-cluster distances (nearest neighbor) were determined by the Euclidean distance from the edge of each cluster to its closest neighboring cluster, with all clusters ≥2 RyR2 channels analyzed. This distance measurement was also used to analyze RyR2 'super-clusters', in which the release of Ca^{2+} from a single cluster would be capable of triggering CICR in a neighboring cluster if located within 150 nm of each other and were subsequently grouped as a functional unit. We opted to use a nearest neighbor distance of <150 nm for super-cluster formation (rather than 100 nm as in previous studies[1, 2]) based on Ca^{2+} modelling carried out in a sheep model of AF.[4] From this, the nearest neighboring distances between super-clusters and the number of clusters within a super-cluster were determined. Generation of the binary mask, as well as the nearest neighbor and super-cluster analyses were performed using custom-written Python scripts. Analyses of RyR2 cluster size and density were performed using ImageJ.

Assessment of RyR2 cluster localization to the z-disk was performed in dual labelled sections for RyR2 super resolution imaging. An image splitter with a dichroic mirror was used to separate the emission onto separate halves of the CMOS camera to enable simultaneous detection of dual labelling, as previously described.[5] Rendered dSTORM images of RyR2 clusters were aligned to the corresponding diffraction-limited (widefield) α-actinin images to provide an accurate indication of z-disk localization. The generated RyR2 masks were overlaid on a binary mask of the corresponding α-actinin labelling to determine the percentage of total clusters aligned to the z-disk (see Supplementary Figure S1). Co-localization analysis was performed based on previously described methods[2, 6] to determine the fraction of total RyR2 labelling associated with the α-actinin labelling, with distances up to and including zero

summed for co-localization, or values up to and including 300 nm summed to determine the fraction of RyR2 labelling within 300 nm of the z-disk.

Supplementary Figures

Figure Legends

Figure S1. Analysis of RyR2 clusters aligning to the z-disk alignment

Image analysis of assessing the alignment of RyR2 cluster to the z-disk involved converting A) the original diffraction-limited image with region of interest (red box) of α-actinin labelling into B) a binary mask based on the location of maximal brightness in the image. C) This mask (green) was then overlaid with the corresponding dSTORM rendered image of RyR2 clusters (red) to determine their localization in relation to the z-disk.

Figure S2. RyR2 cluster size distribution is unchanged in human AF

Accumulated histogram of RyR2 cluster sizes in non-AF (blue), paroxysmal AF (orange) and persistent AF (red) patients determined from dSTORM image analysis.

Figure S3. Overall patient characteristics do not correlate with RyR2 clustering properties

When assessed across the combined patients, correlative analyses revealed no significant relationships between patient age and A) RyR2 cluster size (n=24), B) RyR2 density (n=24) or C) nearest neighbor distance (n=24). Patient RAESV was not significantly correlated with D) RyR2 cluster size (n=20), E) RyR2 density (n=20) or F) nearest neighbor distance (n=20). No significant correlation was observed between patient EF and G) RyR2 cluster size (n=24), H) RyR2 density (n=24) or I) nearest neighbor distance (n=24). All analyses were performed with Pearson's correlation.

Figure S4. Patient characteristics do not correlate with RyR2 clustering properties based on AF status

Correlative analyses were performed to determine differences in the relationships between patient characteristics and RyR2 clustering properties. No significant differences were found between patient age and A) RyR2 cluster size, B) RyR2 density or C) nearest neighbor distance across the three groups. There were no significant differences in patient RAESV correlation with D) RyR2 cluster size, E) RyR2 density or F) nearest neighbor distance between groups. No significant differences in correlation were observed between patient EF and G) RyR2 cluster size, H) RyR2 density or I) nearest neighbor distance. All analyses performed with Pearson's correlation.

Figure S5. Cardiomyocyte size did not correlate with RyR2 clustering properties

When assessed across the combined patients, correlative analyses revealed no significant relationships between cardiomyocyte size and A) RyR2 cluster size, B) RyR2 density or C) nearest neighbor distance. All analyses performed with Pearson's correlation; n=21 per correlation.

Figure S6. Display of nested data points for all patients for RyR2 cluster size and inter-cluster distances

When assess based on hierarchical or nested analyses, no differences were found between groups for A) mean RyR2 cluster size of B) nearest neighbor cluster distances between different patient groups. Data analyzed by nested ANOVA and displayed as mean ± SEM per patient.

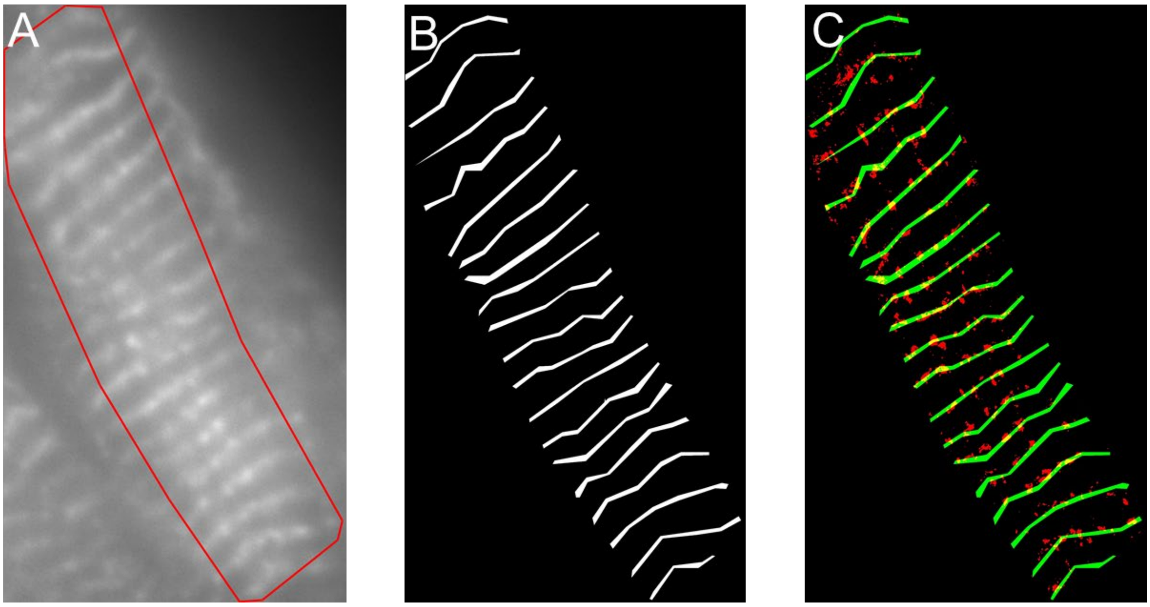

Figure S1. Analysis of RyR2 clusters aligning to the z-disk alignment

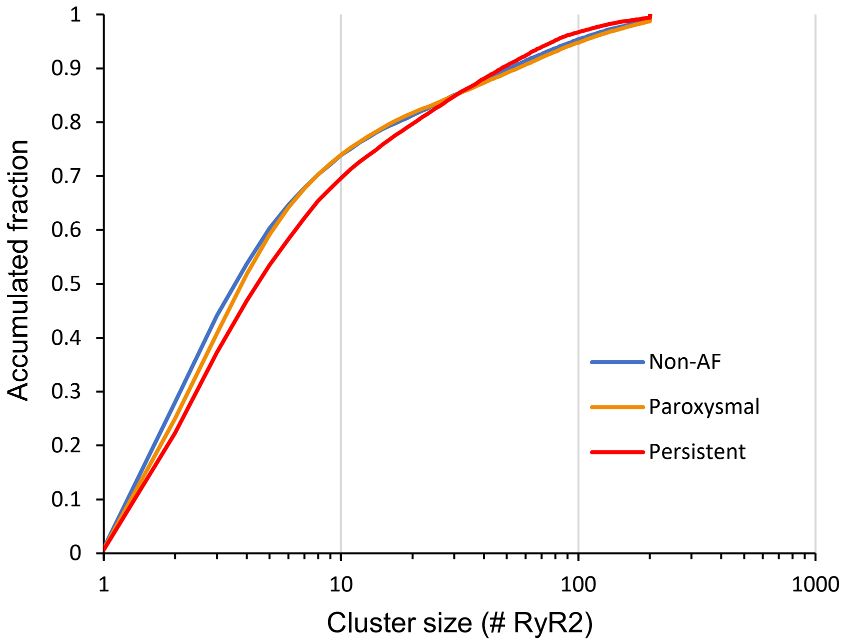

Figure S2. RyR2 cluster size distribution is unchanged in human AF

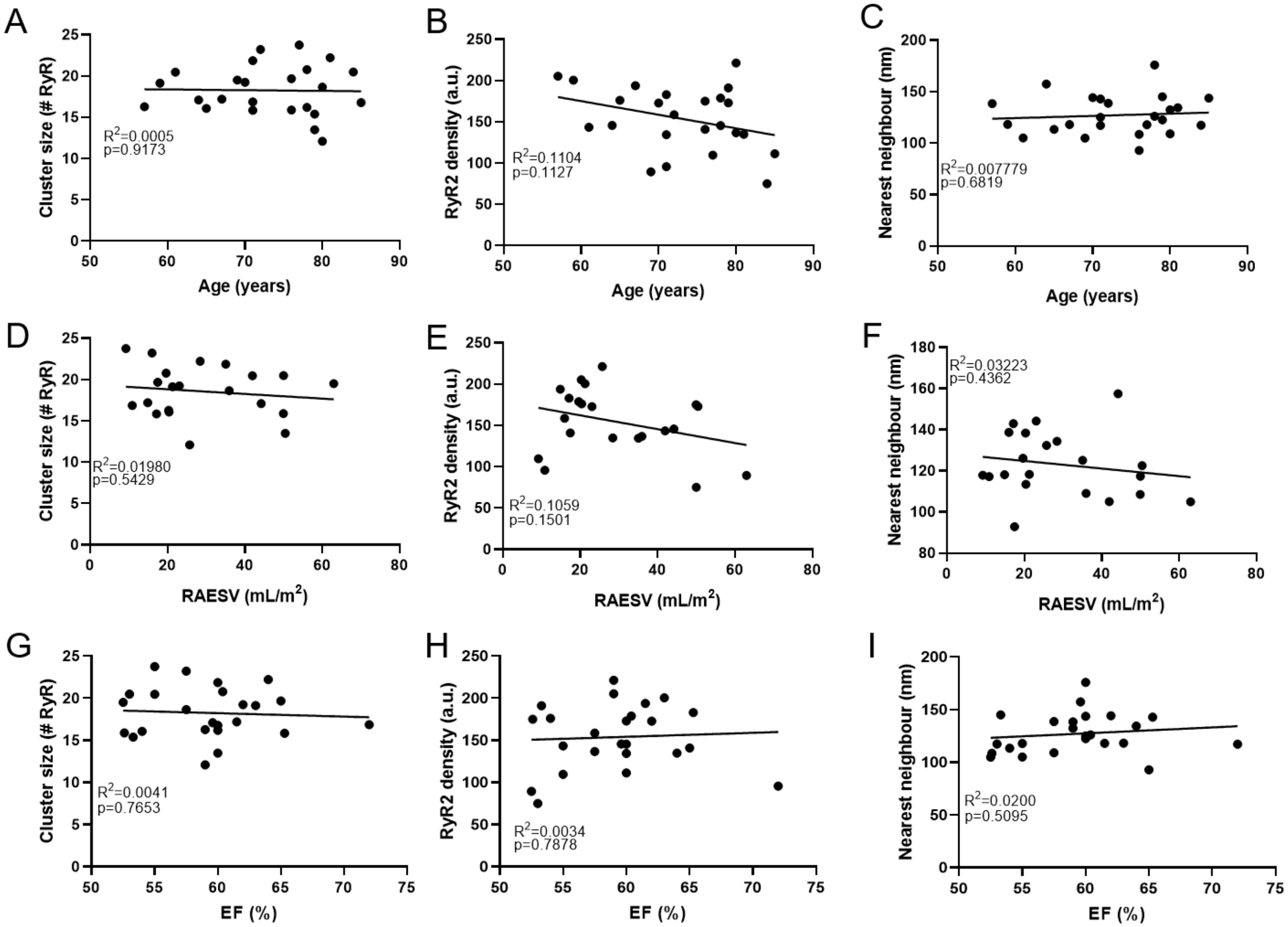

Figure S3. Overall patient characteristics do not correlate with RyR2 clustering properties

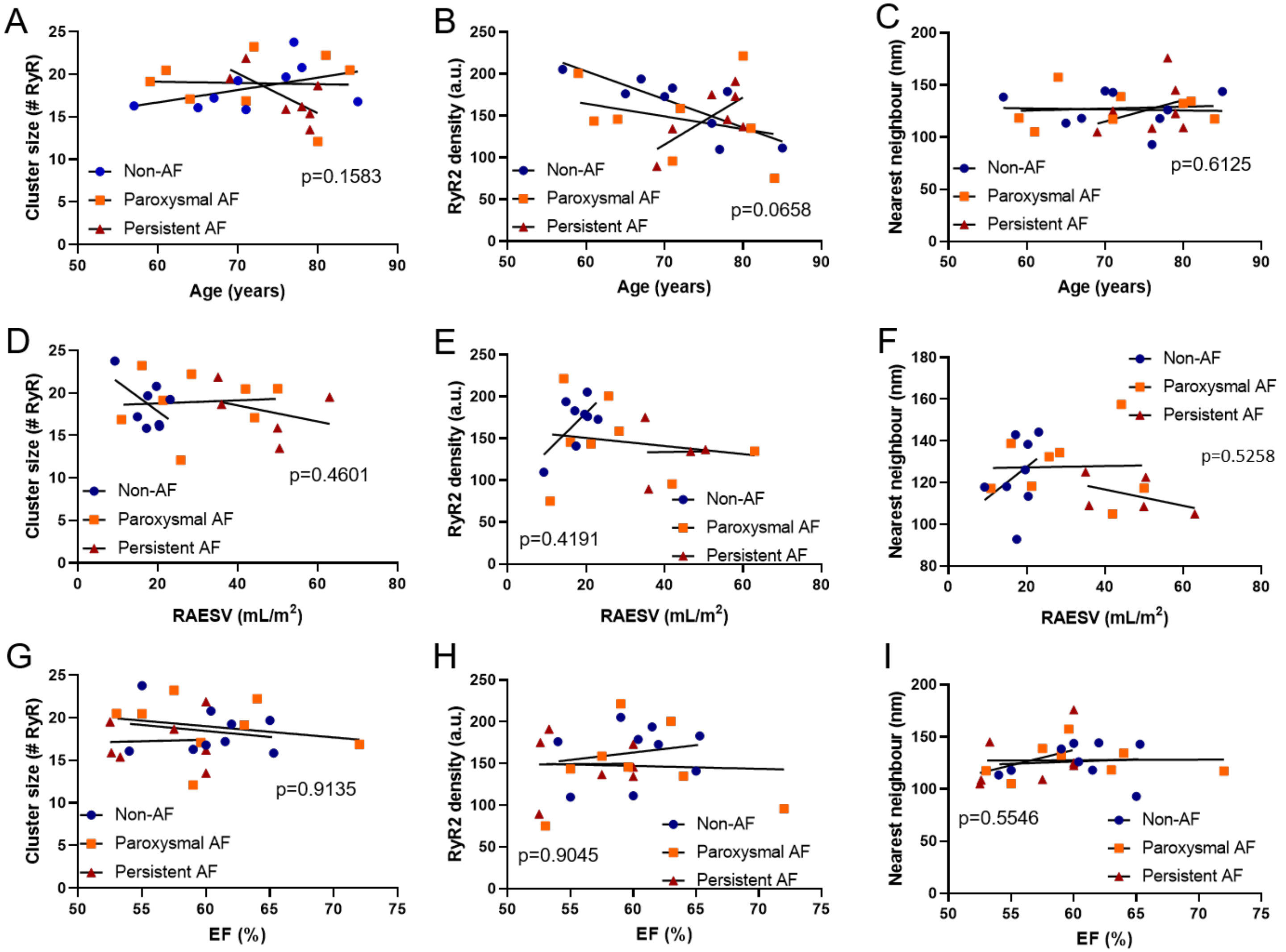

Figure S4. Patient characteristics do not correlate with RyR2 clustering properties based on AF status

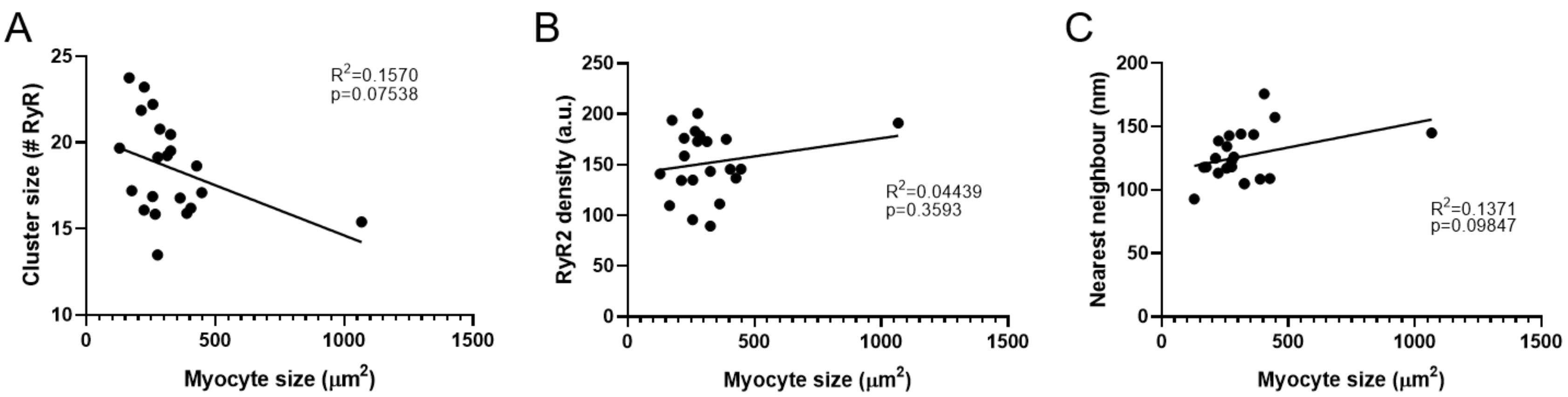

Figure S5. Cardiomyocyte size did not correlate with RyR2 clustering properties

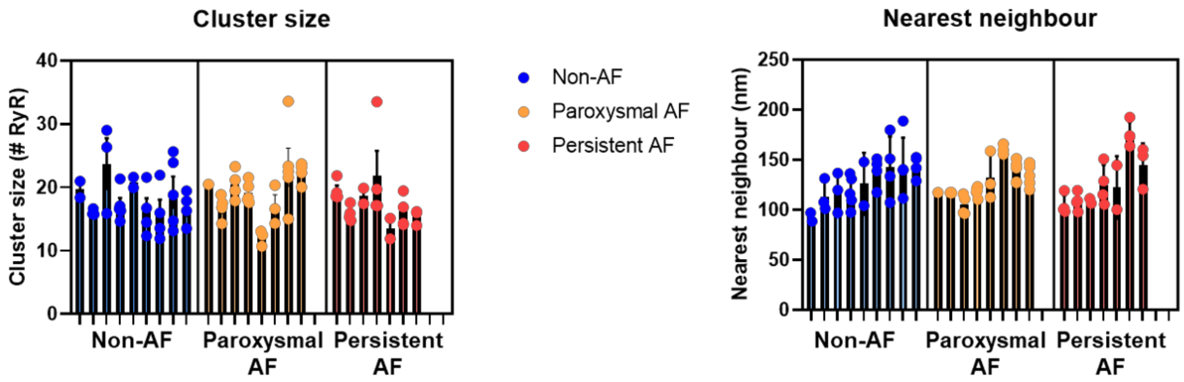

Figure S6. Display of nested data points for all patients for RyR2 cluster size and inter-cluster distances

Supplementary References

1. Hou, Y, Jayasinghe, I, Crossman, DJ, Baddeley, D, Soeller, C: Nanoscale analysis of ryanodine receptor clusters in dyadic couplings of rat cardiac myocytes. *J Mol Cell Cardiol,* 80**:** 45-55, 2015.

2. Munro, ML, Jayasinghe, ID, Wang, Q, Quick, A, Wang, W, Baddeley, D, Wehrens, XH, Soeller, C: Junctophilin-2 in the nanoscale organisation and functional signalling of ryanodine receptor clusters in cardiomyocytes. *J Cell Sci,* 129**:** 4388-4398, 2016.

3. Baddeley, D, Cannell, MB, Soeller, C: Visualization of localization microscopy data. *Microscopy and Microanalysis,* 16**:** 64-72, 2010.

4. Macquaide, N, Tuan, HT, Hotta, J, Sempels, W, Lenaerts, I, Holemans, P, Hofkens, J, Jafri, MS, Willems, R, Sipido, KR: Ryanodine receptor cluster fragmentation and redistribution in persistent atrial fibrillation enhance calcium release. *Cardiovasc Res,* 108**:** 387-398, 2015.

5. Baddeley, D, Crossman, D, Rossberger, S, Cheyne, JE, Montgomery, JM, Jayasinghe, ID, Cremer, C, Cannell, MB, Soeller, C: 4D super-resolution microscopy with conventional fluorophores and single wavelength excitation in optically thick cells and tissues. *PLoS ONE,* 6**:** e20645, 2011.

6. Jayasinghe, ID, Baddeley, D, Kong, Cherrie HT, Wehrens, Xander HT, Cannell, Mark B, Soeller, C: Nanoscale Organization of Junctophilin-2 and Ryanodine Receptors within Peripheral Couplings of Rat Ventricular Cardiomyocytes. *Biophys J,* 102**:** L19-L21, 2012.
